# Limb-Specific Associations Between Isokinetic Knee and Hip Variables and Contact Time Across Sprint Phases in Elite Sprinters

**DOI:** 10.3390/life16101762

**Published:** 2026-10-21

**Authors:** Doga Kural, Berat Turkmen, Hande Argunsah, Turker Sahinkaya, Bulent Bayraktar, Yunus Ziya Arslan

**Affiliations:** 1 Department of Sports Health, Faculty of Sports Science, Istanbul University-Cerrahpasa, 34320 Istanbul, Turkey; doga.kural@iuc.edu.tr (D.K.); berat_trkmn@hotmail.com (B.T.); 2 Department of Biomedical Engineering, Faculty of Engineering and Natural Sciences, Acibadem Mehmet Ali Aydinlar University, 34752 Istanbul, Turkey; 3 Department of Biomedical Engineering, Graduate School of Natural and Applied Sciences, Acibadem Mehmet Ali Aydinlar University, 34752 Istanbul, Turkey; 4 Department of Sports Medicine, Faculty of Medicine, Istanbul University, 34098 Istanbul, Turkey; turkers@istanbul.edu.tr (T.S.); bulent.bayraktar@istanbul.edu.tr (B.B.); 5 Department of Robotics and Intelligent Systems, Institute of Science, Turkish-German University, 34820 Istanbul, Turkey; yunus.arslan@tau.edu.tr

**Keywords:** hip and knee torque, sprint biomechanics, peak torque, high-speed running, limb-specific associations

## Abstract

This study examined exploratory associations between isokinetic knee and hip variables and video-derived contact time across sprint acceleration. Nineteen male sprinters completed 60 m sprints; photocell timing gates recorded sprint times, and eight high-speed cameras operating at 240 frames/s provided contact and flight times. Bilateral concentric knee and hip flexion–extension were assessed at 60°/s and 240°/s for peak torque, relative peak torque, peak power, relative peak power, time to peak torque, and joint angle at peak torque. No statistically significant inter-limb differences were detected in the four torque/power outcomes or mean contact time. Two exploratory knee-extension comparisons at 240°/s yielded nominal raw *p* values below 0.05 for joint angle and time to peak torque, but neither remained statistically significant after Benjamini–Hochberg correction across 16 temporal/angular comparisons. After correction within four complete correlation families, 4 of 26 sprint-time–contact-time correlations remained statistically significant, all involving non-dominant-limb contact time. None of the 832 torque–contact-time, 832 power–contact-time, or 832 time-to-peak-torque/joint-angle-at-peak-torque–contact-time correlations remained significant. The isokinetic correlation patterns are therefore exploratory and require confirmation in larger samples using contact-time methods with established reliability and adequate temporal precision.

## 1. Introduction

Inter-limb strength and power asymmetries in the lower extremities are common in athletes and are increasingly examined for their associations with performance and injury risk [1]. Among the various methods used to assess these asymmetries, isokinetic testing, force platforms, and motion analysis systems are the most widely accepted and frequently utilized tools [2]. While isokinetic testing provides controlled assessments of joint-specific torque production at different angular velocities, force-platform and motion-analysis approaches capture the integrated outcomes of movement during sport-specific tasks [3,4]. Unlike laboratory-based assessments, sprint performance involves the coordinated interaction of multiple joints and muscle groups within a dynamic sporting task [3]. In competition, athletic movements are not constrained to predefined patterns but instead involve high-acceleration sprinting and the attainment of maximal velocity, both of which require rapid force production and precise inter-limb coordination [4,5,6]. During sprinting, performance is strongly influenced by temporospatial parameters such as contact time (CT), flight time (FT), and step frequency, as well as by the ability to generate force at high angular velocities in the lower limbs [7,8]. Therefore, understanding how laboratory-based strength characteristics relate to sprint-specific mechanical demands remains an important challenge in sports biomechanics [9].

Although isokinetic measures have demonstrated associations with sprint and jump performance, strength asymmetries identified under controlled laboratory conditions may not necessarily manifest as functional asymmetries during sprinting [1,10,11]. Compared with field-based functional assessments, isokinetic testing enables precise and isolated torque measurements at controlled angular velocities, allowing inter-limb differences to be quantified without the influence of sport-specific movement variability [11,12,13,14,15,16]. Moreover, isokinetic measures have demonstrated high predictive value for sport-specific performance [17,18,19]. However, how between-limb differences in isokinetic strength relate to limb-specific functional characteristics during sprinting remains unclear.

Elite sprinters typically cover 30 m in approximately 3.60–3.90 s [20,21], although the point at which maximal velocity is reached varies according to individual acceleration patterns [5,20]. During sprint acceleration, running velocity progressively increases, accompanied by reductions in CT and increases in lower-limb joint angular velocities [22,23,24,25,26]. Consequently, the mechanical demands of sprinting differ across acceleration phases, with early phases characterized by longer CT and lower movement velocities, whereas later phases require rapid force production at substantially higher angular velocities [5,27]. Accordingly, the relationship between isokinetic strength and sprint performance may be phase-specific and dependent on the angular velocity at which strength is assessed [28,29].

Additionally, İnce and Tortu (2025) examined inter-limb and agonist–antagonist isokinetic knee torque asymmetries alongside jump and sprint tests and found only low correlations between inter-limb torque asymmetry and sprint or jump power measures [30]. Coratella et al. (2018) reported moderate correlations between concentric hamstring peak torque asymmetry at 300°/s and sprint performance in football players [31]. However, both studies primarily focused on overall sprint outcomes and did not examine limb-specific temporospatial characteristics during sprinting. Similarly, Exell et al. (2017) identified significant inter-limb differences in kinetic and spatiotemporal sprint variables during maximal-velocity running [28]. However, their analysis was limited to a small number of steps collected at maximal speed and did not investigate associations with isokinetic strength measures. Consequently, despite growing interest in lower-limb asymmetries, evidence linking limb-specific sprint mechanics with isokinetic strength characteristics remains limited.

Building on these findings, inter-limb asymmetries have commonly been investigated using isolated outcome measures, such as isokinetic strength assessments and sprint spatiotemporal variables. Likewise, numerous studies have examined relationships between lower-limb strength and sprint performance. Nevertheless, it remains unclear whether the magnitude of between-limb differences in isokinetic strength is associated with limb-specific sprint mechanics, particularly with respect to contact-time characteristics across different acceleration phases.

Therefore, the primary outcome of this study was video-derived contact time (CT) across predefined phases of a 60 m sprint. The primary hypothesis was that isokinetic knee and hip torque and power variables, particularly those assessed at 240°/s, would be associated with CT in a phase-specific manner, reflecting the velocity-dependent mechanical demands of sprint acceleration [7,32]. The prespecified correlation analyses examined (i) the relationships between sprint interval times and CT and (ii) the associations of peak torque, relative peak torque, peak power, and relative peak power at 60°/s and 240°/s with CT across sprint phases. Flight time and linear velocity were treated descriptively. Comparisons of association patterns according to limb classification, including ipsilateral and contralateral relationships, together with the analyses involving time to peak torque and joint angle at peak torque, were considered exploratory.

## 2. Materials and Methods

The present study was designed to investigate whether laboratory-based between-limb differences in lower-limb strength are associated with limb-specific contact-time characteristics during sprinting. To minimize fatigue effects and ensure independent assessment of each performance domain, isokinetic and sprint testing sessions were conducted on separate days with a maximum interval of 48 h. The study consisted of two complementary data collection procedures: (1) isokinetic testing of the hip and knee joints to quantify torque and power at both low and high angular velocities, and (2) sprint time and motion analysis to determine CT and FT across a 60 m sprint.

This experimental design allowed for the integration of isolated strength measures obtained under controlled laboratory conditions with kinematic and temporospatial parameters measured in a realistic sprint environment, providing a comprehensive evaluation of between-limb differences in strength and movement performance.

### 2.1. Subjects

A total of 26 male sprinters initially participated in this study. However, seven athletes were excluded for the following reasons: one athlete sustained an ankle sprain between the isokinetic and sprint measurement sessions; three athletes exceeded the allowable 48 h interval between the two test sessions; and motion capture errors occurred in the sprint video recordings of three athletes. Consequently, data from 19 male sprinters were included in the final analysis. The participants specialized in the 60 m, 100 m, 200 m, and 400 m sprint events, with five participants competing in sprint hurdles (60 m, 110 m, or 400 m hurdles). They had a mean age of 20.95 ± 3.36 years, a mean height of 180.89 ± 6.51 cm, and a mean body weight of 72.95 ± 7.31 kg. Dominance was determined using the athlete’s self-reported preferred kicking leg, a commonly used criterion in sport-science research to classify functional limb laterality [33]. Ethical approval was granted by the Institutional Review Board. All procedures were carried out in accordance with the principles outlined in the Declaration of Helsinki, and written informed consent was obtained from all participants.

To justify the classification of the participants as elite sprinters, all athletes previously represented their national teams at least once at youth or senior levels (U17, U18, U20/U23, or senior categories). In addition, all participants had a minimum of six years of systematic sprint-training experience. The athletes’ individual 100 m personal-best performances averaged 10.94 ± 0.33 s and ranged from 10.24 to 11.42 s, supporting the characterization of the sample as highly trained competitive sprinters.

### 2.2. Procedures

The 60 m sprint tests were conducted on a World Athletics-certified tartan track at the Athletics Federation Sports Arena. Timing gates were positioned at 0, 5, 10, 20, 30, 45, and 60 m and set at a height of 1.0 m to record sprint times across the acceleration phases using the Newtest Powertimer 300 photocell timing system (Newtest Oy, Finland), with data recorded and analyzed using Newtest PowertimerPC 2.0 software for Windows. The starting line was positioned 0.5 m behind the first timing gate to prevent premature triggering of the system. Athletes started from starting blocks and individually adjusted the front and rear block positions according to their habitual settings. The starting procedure consisted of the verbal commands “On your marks” and “Set,” followed by a hand clap as the start signal. Electronic timing commenced when the athlete crossed the first timing gate. All athletes wore their own sprint spikes.

Eight high-speed cameras (GoPro 8 Black, 1920 × 1080 px, 240 fps) were positioned bilaterally at approximately 1.2–1.5 m (pelvis level) and 8.5 m from the running lane to optimize the capture of step-specific contact and flight events during sprinting [34]. Four successive recording zones covered approximately 0–15, 15–30, 30–45, and 45–60 m on each side of the lane. Each camera covered approximately 18 m of the running lane, producing an overlap of approximately 2 m between adjacent recording zones and allowing contacts to be followed across camera transitions. The cameras were set at 240 fps with a shutter speed of 1/480 s to minimize motion blur and facilitate the identification of touchdown and toe-off events.

Before testing, participants performed a standardized warm-up including jogging, static and dynamic stretching, and submaximal runs. Each participant completed three maximal sprint trials with 5 min rest intervals, and the trial with the shortest 60 m sprint time was selected for analysis [35,36]. The fastest trial was selected a priori on the basis of total 60 m sprint time and before the frame-by-frame extraction of CT and FT. This approach was used to characterize the contact-time profile associated with each athlete’s best realized maximal-effort sprint performance and to ensure that the sprint-time and video-derived variables were obtained from the same trial, rather than averaging observations across trials with potentially different phase-specific performance profiles. Motion-capture data were analyzed using Kinovea 9.5.0 to determine CT and FT through frame-by-frame identification of touchdown and toe-off events. The first complete ground contact after the athlete crossed the initial timing gate was included in the analysis. Contacts were assigned to the relevant sprint interval according to the spatial location of the complete stance phase from touchdown to toe-off. A contact completed before an interval boundary was assigned to the preceding interval, whereas the first complete contact beyond that boundary was assigned to the subsequent interval.

When multiple contacts from the same limb occurred within a predefined sprint interval, CT values were averaged separately for the dominant and non-dominant limbs, yielding one mean CT value for each limb, athlete, and interval. Event-level measurements obtained from the successive camera zones were pooled in spatial order across the 60 m sprint. When the same contact was visible in the overlapping fields of two adjacent cameras, it was included only once to prevent duplication. At the recording frequency of 240 fps, one frame represented approximately 4.17 ms; therefore, uncertainty of one frame at either event boundary could affect the derived CT by approximately 4.17 ms, with a potentially greater cumulative effect if both boundaries were uncertain. Because the analysis was based on temporal frame counts rather than spatial measurements, no spatial calibration was required, and no signal-filtering procedure was applied to these discrete frame-derived temporal variables.

All analyses were performed by the same experienced evaluator. CT and FT were initially recorded for the right and left limbs and classified as dominant or non-dominant only after data collection. Uncertain touchdown or toe-off events were reviewed before final frame selection; however, this quality-control procedure did not constitute a formal intra-rater reliability assessment. The original protocol did not include duplicate ratings, and the retained analytical dataset contained only one finalized value for each event; consequently, formal intra-rater or inter-rater reliability estimates were not available.

Isokinetic strength assessments were performed using the HUMAC Norm Cybex dynamometer to measure Peak Torque (PT), Relative Peak Torque (RPT), Peak Power (PP), Relative Peak Power (RPP), Time to Peak Torque (TTPT), and Joint Angle at Peak Torque (JAPT) during concentric flexion and extension of the knee and hip joints for both non-dominant (NDL) and dominant limbs (DL). Relative peak torque was calculated by dividing peak torque (Nm) by body mass (kg) and was expressed as Nm·kg^−1^. Similarly, relative peak power was calculated by dividing peak power (W) by body mass (kg) and was expressed as W·kg^−1^. Prior to testing, the dynamometer was calibrated according to the manufacturer’s recommendations (HUMAC Norm/Cybex protocol) with no attachment connected to the lever arm. All knee and hip assessments were performed through a 100° range of motion using concentric flexion and extension contractions. For knee testing, the anatomical zero position (full knee extension, 0°) was first established, and the range of motion extended from 0° to 100° knee flexion. For hip testing, the neutral anatomical position (legs aligned) served as the reference position, and the range of motion extended from 0° to 100° of hip flexion. Gravity correction was performed for all participants according to the HUMAC Norm protocol. During knee testing, participants were instructed to completely relax the tested limb at full knee extension while the dynamometer measured the gravitational moment (Nm), which was automatically incorporated into both flexion and extension torque calculations. During hip testing, gravity correction was performed with the hip positioned at 45° of flexion following the manufacturer’s protocol.

For each participant and condition, inter-limb asymmetry was quantified using the conventional percentage-difference formula, |DL − NDL|/max(DL, NDL) × 100, which provides a standardized unsigned measure of the relative magnitude of the between-limb difference [11,37,38]. Condition-specific asymmetry-index values were summarized as the mean of the individual participant values and were used descriptively only; paired inference was based on signed within-participant differences. Knee testing was conducted in a seated position, while hip testing was performed in the supine position, with all alignments set according to the manufacturer’s guidelines to ensure anatomical accuracy. Participants were stabilized using the manufacturer’s restraint system. During knee testing, straps secured the trunk, pelvis, and tested thigh, while the contralateral lower limb was stabilized to minimize unwanted movements. During hip testing, the trunk and pelvis were similarly stabilized, and the contralateral limb was secured using the manufacturer’s fixation straps.

Participants completed a warm-up consisting of treadmill walking and lower-limb stretching before testing [39,40]. Assessments were conducted at 60°/s (three maximal repetitions) and 240°/s (five maximal repetitions), with submaximal practice trials preceding each velocity condition to ensure familiarization [40,41]. Standardized verbal encouragement (“Push strong”, “Pull strong”, “Push faster”, and “Pull faster”) was provided throughout all maximal efforts, whereas visual feedback from the dynamometer monitor was not provided. The coefficient of variation (CV) across maximal repetitions was monitored as a quality-control indicator. A CV below 10% was used as the predefined target for repetition consistency rather than as an absolute exclusion criterion. CV values ranged from 2% to 9% for the knee assessments and from 4% to 15% for the hip assessments. Hip test sets exceeding the 10% target were qualitatively reviewed for irregularities in the torque curves and for possible errors in movement execution before being retained for analysis.

Testing began with the DL, followed by the NDL after a three-minute rest, and the knee was tested before the hip in all participants [42,43]. Rest intervals were provided between conditions to prevent fatigue and ensure the reliability of measurements. For each movement direction and angular-velocity condition, all outcome variables were first determined separately for each valid repetition and were then averaged across the three repetitions performed at 60°/s or the five repetitions performed at 240°/s using the HUMAC Norm (version 2015) software. Specifically, PT represented the maximum torque attained during each repetition, while PP represented the maximum power attained during each repetition. RPT and RPP were obtained by normalizing the corresponding repetition-specific PT and PP values to body mass. TTPT was defined as the elapsed time from movement onset to the occurrence of PT within each repetition, and JAPT represented the joint angle at which PT occurred. The repetition-specific values for PT, RPT, PP, RPP, TTPT, and JAPT were subsequently averaged to obtain one value for each variable, limb, joint action, and angular-velocity condition for each participant. This approach was adopted to provide a more representative estimate of each participant’s performance by reducing the influence of trial-to-trial variability. Furthermore, because sprint performance requires repeated force production across consecutive ground contacts rather than a single isolated maximal effort, the mean value was considered more representative of the functional demands of sprinting than a single peak repetition.

### 2.3. Statistical Analysis

The primary statistical analyses were performed using IBM SPSS Statistics version 24.0 (IBM Corp., Armonk, NY, USA). Given the limited availability of directly comparable empirical estimates on which to base an expected correlation, the statistical sensitivity of the achieved sample was evaluated using G*Power software (version 3.1.9.4, Heinrich Heine University Düsseldorf, Düsseldorf, Germany) for a two-tailed bivariate correlation model. With 19 participants, an alpha level of 0.05, and 80% statistical power, the minimum detectable correlation was |ρ| = 0.59. This threshold represents a large correlation according to conventional effect-size benchmarks [44], indicating that the study was primarily capable of detecting large associations and had limited power to identify moderate correlations. Accordingly, non-significant correlations, including those involving variables assessed at 60°/s, were interpreted as an absence of statistically detectable associations rather than evidence that no relationship exists. The normality of the paired differences was assessed using the Shapiro–Wilk test. Paired-samples *t*-tests were used to compare isokinetic variables and CT between the DL and NDL. Two-tailed Pearson correlations were calculated for (i) each phase-matched sprint-time–CT pair and (ii) the associations between the isokinetic parameters (PT, RPT, PP, RPP, TTPT, and JAPT) and limb-specific CT across sprint phases. The isokinetic–CT analyses included both ipsilateral (same-limb) and contralateral (cross-limb) combinations.

The prespecified analytical expectation was that isokinetic variables assessed at 240°/s would exhibit correlation coefficients of greater absolute magnitude than variables assessed at 60°/s. However, correlation coefficients were not formally compared between angular velocities or limb classifications. Therefore, any observed differences in correlation magnitude were interpreted descriptively and were not considered evidence that the corresponding correlations differed statistically. Analyses involving limb-specific and cross-limb relationships, as well as TTPT and JAPT variables, were treated as exploratory. Accordingly, findings derived from these exploratory analyses were interpreted with appropriate caution.

Because several cumulative and segmental sprint intervals overlapped and consequently contained some of the same ground contacts, correlations across these intervals were not considered statistically or biomechanically independent. For biomechanical interpretation, five non-overlapping phases (0–5, 5–20, 20–30, 30–45, and 45–60 m) were used as reference phases where applicable, representing initial, early, mid-, and late acceleration and the transition toward near-maximal velocity. However, every association that survived FDR correction in the complete 26-test sprint-time–CT family was reported in the main text regardless of interval overlap. The remaining cumulative or overlapping results were treated as supplementary and exploratory. All eligible correlations from both overlapping and non-overlapping intervals were retained within their respective Benjamini–Hochberg families to avoid result-dependent selection of tests.

The directionality of correlations was interpreted within the context of performance outcomes. For example, a positive correlation between sprint time and CT indicates a performance decline (i.e., longer ground contact is associated with slower sprinting), whereas a negative correlation between isokinetic torque or power and CT suggests improved performance (i.e., higher torque is associated with shorter CT, reflecting more efficient force production during sprinting). It is therefore important to note that negative correlations in this context indicate favorable performance characteristics rather than detrimental effects. Given that joint torque and power demands vary according to angular velocities and across different sprint phases, both positive and negative correlations were anticipated. Statistical significance thresholds were defined as *p* < 0.05 (*), *p* < 0.01 (**), *p* < 0.001 (***).

For the primary PT/RPT/PP/RPP paired comparisons, Cohen’s dz effect sizes and 95% confidence intervals for the mean differences were calculated and are presented in Table 1. To address multiplicity in the exploratory temporal/angular comparisons, the 16 paired-test *p* values for TTPT and JAPT (2 joints × 2 movement directions × 2 angular velocities × 2 variables) were entered into a separate Benjamini–Hochberg procedure; complete results are provided in Table S1. For the correlation analyses, the Benjamini–Hochberg false discovery rate (FDR) procedure was applied separately to four domain-based families: (1) sprint-time–CT correlations, comprising 26 tests; (2) torque–CT correlations involving PT and RPT, comprising 832 tests; (3) power–CT correlations involving PP and RPP, comprising 832 tests; and (4) TTPT/JAPT–CT correlations, comprising 832 tests. Each isokinetic family included all combinations of joint, movement direction, angular velocity, isokinetic limb, sprint phase, and CT limb. All eligible correlations were entered into the Benjamini–Hochberg procedure irrespective of their nominal statistical significance. Because these correlation FDR families were not fully specified prospectively, the correlation analyses were interpreted as exploratory. The complete correlation matrix, including r, raw *p*, adjusted *p*, and 95% confidence intervals, is provided in Table S1.

To assess the robustness of the FDR-significant sprint-time–CT findings in this small sample, all 26 sprint-time–CT associations were recomputed using Spearman’s rank-order correlation and subjected to the same Benjamini–Hochberg correction. Potentially influential observations were evaluated in the four FDR-significant Pearson models using Cook’s distance, with 4/*n* used as a screening threshold. Leave-one-out sensitivity analyses were also performed by excluding each participant in turn and recalculating all 26 Pearson correlations and their corresponding Benjamini–Hochberg-adjusted *p*-values. These sensitivity analyses were performed using Python version 3.12.14 with NumPy version 2.3.5 and SciPy version 1.17.0.

## 3. Results

**Figure 1:**
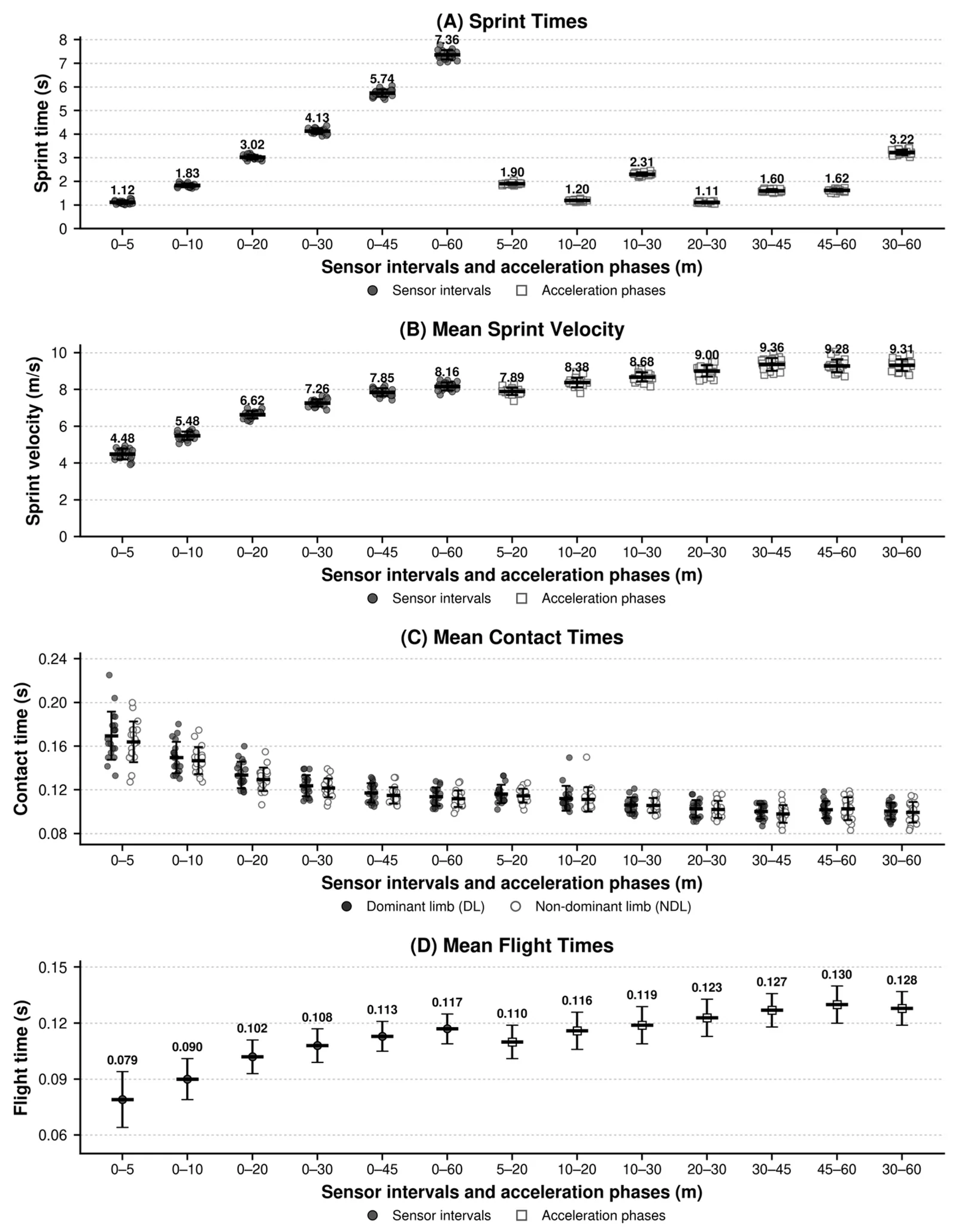
Individual participant values and mean ± SD for sprint times (**A**), interval-average velocities (**B**), and contact times for the dominant and non-dominant limbs (**C**); mean ± SD flight times are shown in panel (**D**). Filled circles indicate cumulative sensor intervals and open squares indicate segmental acceleration phases in panels (**A**,**B**,**D**).

All 19 sprinters successfully completed their maximal-effort 60 m sprint trials without any complications. Sprint times and velocities were recorded across sensor-defined cumulative intervals (0–5 m, 0–10 m, 0–20 m, 0–30 m, 0–45 m, and 0–60 m) and additional acceleration-phase segments (5–20 m, 10–20 m, 10–30 m, 20–30 m, 30–45 m, 45–60 m, and 30–60 m), which were defined before the statistical analyses to characterize sprint acceleration and the transition toward near-maximal velocity based on previous sprint-biomechanics literature and the temporal resolution provided by the seven-photocell system [22,45]. Segmental times were derived from the cumulative timing-gate values. Cumulative intervals were used to characterize overall sprint progression, whereas segment-specific intervals were included to examine phase-specific changes. Sprint times and interval-average velocities are presented in Figure 1A,B; CT and FT summaries are shown in Figure 1C,D.

### 3.1. Sprint Performance and Contact-Time Characteristics

The sprinters completed the 60 m distance in 7.36 ± 0.20 s, and their highest interval-average velocity was 9.36 ± 0.35 m/s, observed over the 30–45 m interval. The longest CT values were recorded in the initial 0–5 m segment, measured at 0.169 ± 0.022 s for the DL and 0.164 ± 0.019 s for the NDL, reflecting the demands of initial acceleration. In contrast, the shortest CT values occurred in the 30–45 m interval, with 0.100 ± 0.007 s for the DL and 0.098 ± 0.008 s for the NDL, consistent with the attainment of near-maximal sprint velocities (Figure 1).

There were no statistically significant differences between the dominant (DL) and non-dominant (NDL) limbs in contact time across any sprint segment (all *p* > 0.05). Descriptive percentage differences between limbs ranged from approximately 0% to 3% across the sprint phases. Given the measurement considerations described in Section 2.2, these differences were interpreted descriptively and were not considered evidence of either true inter-limb contact-time asymmetry or bilateral equivalence. Contact time (CT) was the prespecified temporal variable for the inferential analyses, whereas flight time (FT) values are presented descriptively.

### 3.2. Inter-Limb Comparisons of Isokinetic Variables

**Figure 2:**
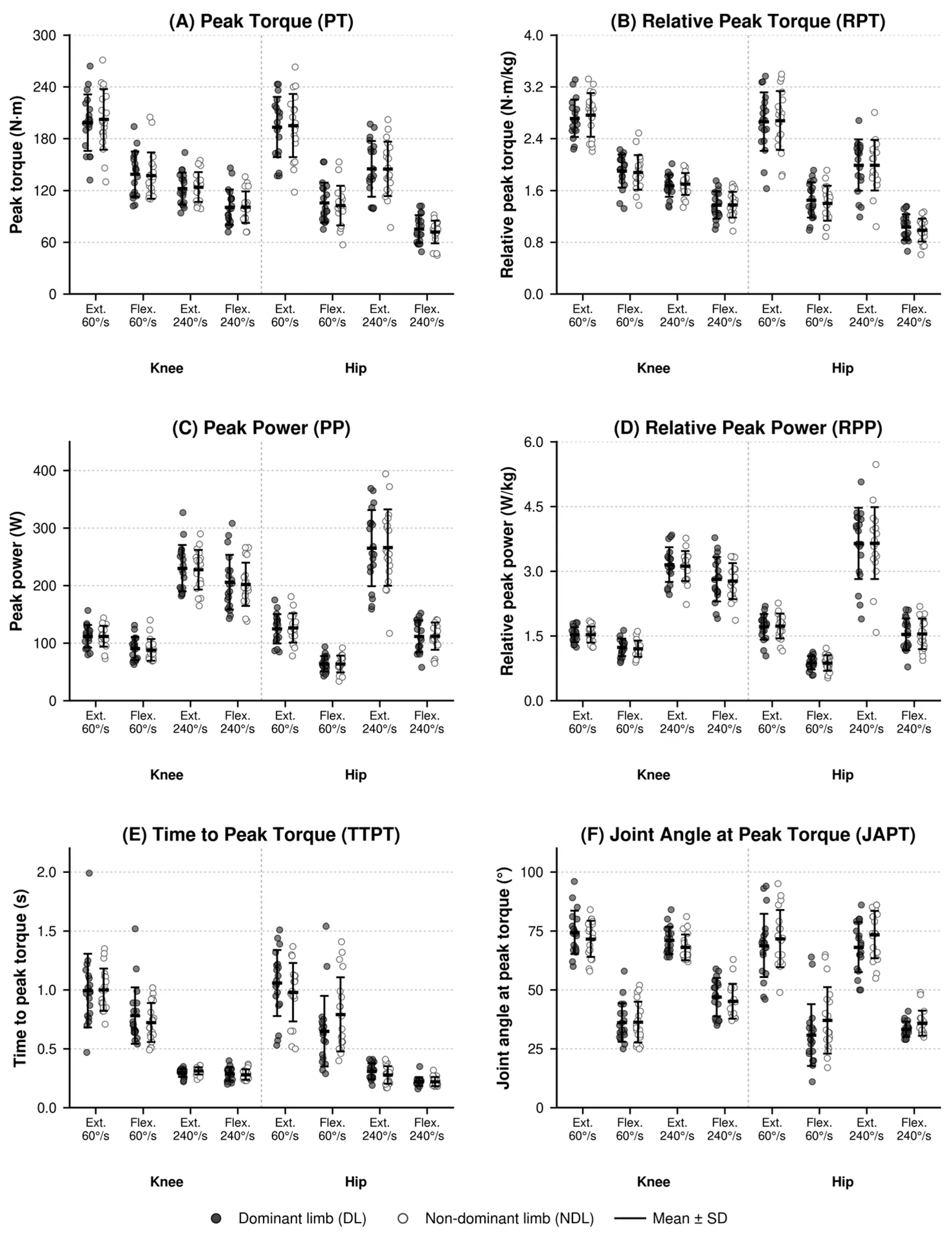
Individual participant values and mean ± SD for inter-limb comparisons of isokinetic variables for the knee and hip joints: peak torque (PT) and relative peak torque (RPT) (**A**,**B**), peak power (PP) and relative peak power (RPP) (**C**,**D**), and time to peak torque (TTPT) and joint angle at peak torque (JAPT) (**E**,**F**) at 60°/s and 240°/s. Filled circles represent the dominant limb (DL), open circles represent the non-dominant limb (NDL), and horizontal bars with error bars represent mean ± SD.

Peak torque (PT) and relative peak torque (RPT) values at 60°/s and 240°/s for knee and hip flexion and extension are illustrated in Figure 2A,B. No statistically significant systematic inter-limb differences were detected in PT or RPT across any movement direction or angular velocity (all *p* > 0.05; Table 1). Across the PT and RPT conditions, the condition-specific means of the participant-level asymmetry indices ranged from 5.31% to 12.50%.

No statistically significant systematic inter-limb differences were detected in PP or RPP at either angular velocity (all *p* > 0.05; Figure 2C,D and Table 1); mean participant-level asymmetry indices across the PP and RPP conditions ranged from 6.46% to 14.16%. For the exploratory temporal/angular outcomes, two knee-extension comparisons at 240°/s had nominal raw *p* values below 0.05. JAPT was 71.0 ± 5.8° for the DL and 68.1 ± 5.5° for the NDL (mean difference = 2.95°, 95% CI 0.95 to 4.94, Cohen’s dz = 0.711, raw *p* = 0.006), while TTPT was 0.30 ± 0.04 s for the DL and 0.31 ± 0.03 s for the NDL (mean difference = −0.016 s, 95% CI −0.028 to −0.003, Cohen’s dz = −0.605, raw *p* = 0.017). Neither comparison remained statistically significant after Benjamini–Hochberg correction across the 16 TTPT/JAPT comparisons (adjusted *p* = 0.099 and 0.134, respectively). All other raw TTPT/JAPT *p* values were ≥0.050. Mean participant-level asymmetry indices ranged from 7.80% to 29.12% for TTPT and from 5.92% to 25.54% for JAPT. Descriptive mean ± SD values for all isokinetic variables and complete TTPT/JAPT paired comparisons are reported in Table S2.

The asymmetry index is an unsigned participant-level magnitude and is therefore not directly comparable with the signed group-level mean difference from a paired test. Neither the descriptive asymmetry indices nor non-significant paired comparisons demonstrate bilateral symmetry or equivalence, and they do not exclude larger inter-limb differences in individual athletes. Primary PT/RPT/PP/RPP paired statistics are summarized in Table 1, whereas the exploratory TTPT/JAPT comparisons are reported in Table S2.

### 3.3. Exploratory Limb-Specific Associations Between Isokinetic Variables and Sprint Contact Time

**Figure 3:**
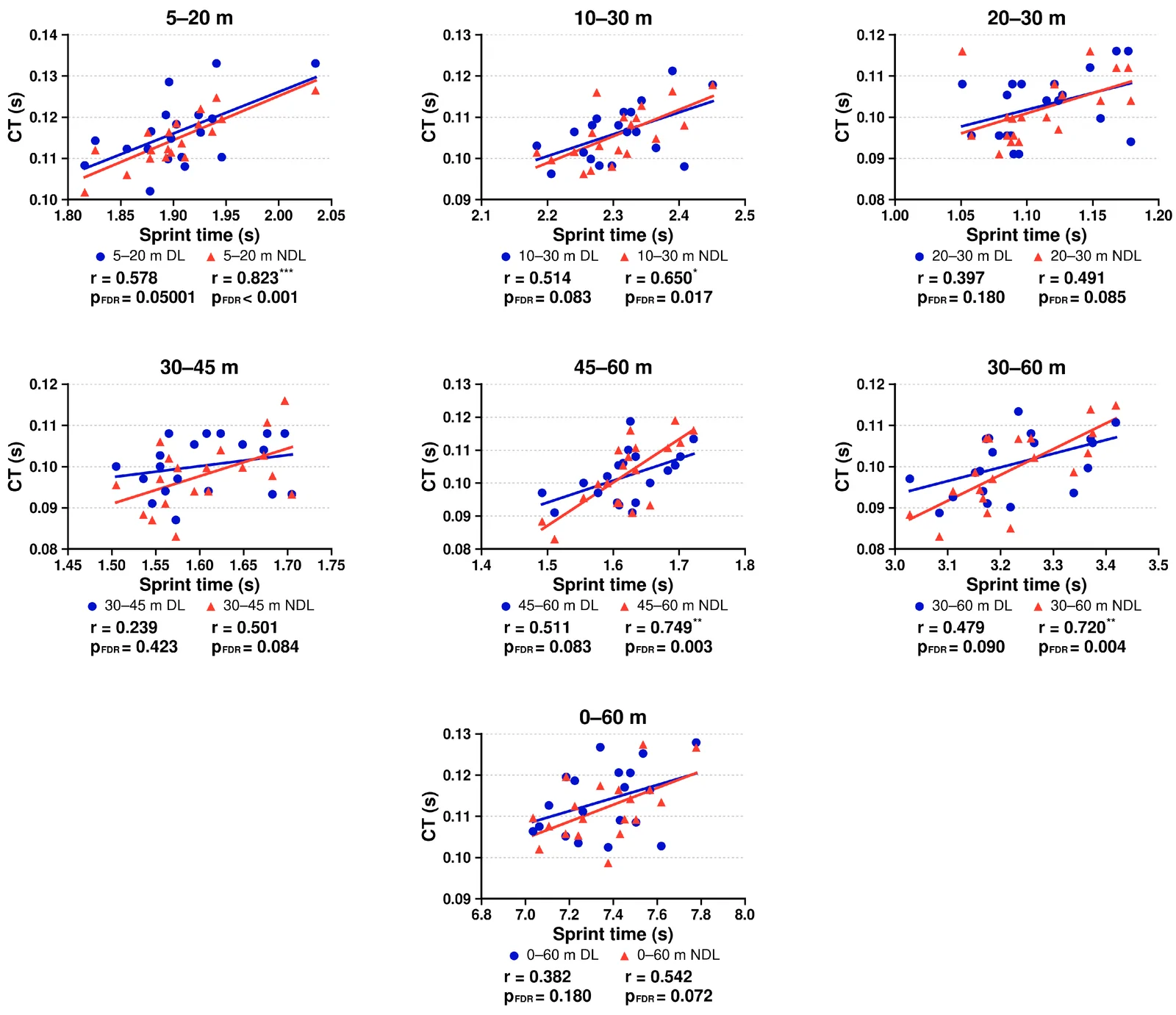
Selected correlations between sprint interval times and dominant-limb (DL) and non-dominant-limb (NDL) contact time (CT). The panels display the seven sprint intervals in which at least one DL or NDL correlation had a nominal raw *p* value below 0.05. Pearson correlation coefficients (r) are shown together with Benjamini–Hochberg FDR-adjusted *p* values (*p*_FDR) calculated across the complete 26-test sprint-time–CT family. Significance symbols are based exclusively on the adjusted *p* values (* *p*_FDR < 0.05, ** *p*_FDR < 0.01, and *** *p*_FDR < 0.001). Raw *p* values and the complete correlation results are provided in Table S1.

No statistically significant systematic DL–NDL differences were detected for PT, RPT, PP, RPP, or mean CT; the two nominal knee-extension TTPT/JAPT findings at 240°/s also did not remain statistically significant after multiplicity correction. The exploratory correlation analyses nevertheless showed different numerical patterns according to limb classification. Correlations between sprint interval times and limb-specific CT are presented in Figure 3. Before correction for multiple testing, 11 of the 26 sprint-time–CT correlations had raw *p* values below 0.05. After all 26 correlations were entered into the Benjamini–Hochberg procedure, four associations remained statistically significant, all involving NDL CT: 5–20 m (r = 0.823, raw *p* < 0.001, adjusted *p* < 0.001), 10–30 m (r = 0.650, raw *p* = 0.003, adjusted *p* = 0.017), 30–60 m (r = 0.720, raw *p* < 0.001, adjusted *p* = 0.004), and 45–60 m (r = 0.749, raw *p* < 0.001, adjusted *p* = 0.003). No correlation involving DL CT remained statistically significant after FDR correction. However, the DL and NDL correlation coefficients were not formally compared, and differences between them should therefore be interpreted descriptively. The FDR-significant correlations are summarized in Table 2, and the complete results are provided in Table S1.

For descriptive visualization, Figure 3 presents the seven sprint intervals in which at least one correlation involving DL or NDL CT had a nominal raw *p* value below 0.05; collectively, these panels contain all 11 nominal associations identified within the complete 26-test sprint-time–CT family. The cumulative and segmental intervals were retained to provide complementary temporal perspectives across sprint acceleration and overall sprint performance; however, associations observed across overlapping intervals were not interpreted as independent replications.

Spearman sensitivity analysis reproduced the four FDR-significant Pearson associations, all involving NDL CT: 5–20 m (ρ = 0.795, adjusted *p* = 0.001), 10–30 m (ρ = 0.677, adjusted *p* = 0.010), 30–60 m (ρ = 0.735, adjusted *p* = 0.004), and 45–60 m (ρ = 0.672, adjusted *p* = 0.010). One additional Spearman-only association was identified for 10–20 m segment time and NDL CT (ρ = 0.639, raw *p* = 0.003, adjusted *p* = 0.017); the corresponding Pearson result was not statistically significant (r = 0.385, raw *p* = 0.104, adjusted *p* = 0.180). Cook’s distance screening identified two potentially influential observations for the 5–20 m association (D > 4/n) and none for the other three Pearson models. In leave-one-out analyses, Pearson coefficients remained positive and statistically significant at the unadjusted level for all four associations. After recalculating the 26-test FDR family in each leave-one-out dataset, the 5–20, 30–60, and 45–60 m associations remained FDR-significant in all 19 iterations, whereas the 10–30 m association remained FDR-significant in 18 of 19 iterations. Thus, the four Pearson findings were generally robust to the sensitivity analyses performed, although the 10–30 m result showed some sensitivity to individual observations; the additional 10–20 m finding was specific to the rank-based analysis. Full sensitivity results are provided in Table S3.

Before FDR correction, 42 of the 832 torque–CT correlations, 52 of the 832 power–CT correlations, and 31 of the 832 TTPT/JAPT–CT correlations had nominal raw *p* values below 0.05. However, none remained statistically significant after Benjamini–Hochberg correction (minimum adjusted *p* = 0.633, 0.741, and 0.865, respectively). These analyses should therefore be interpreted as exploratory. Family-level results are summarized in Table 3, and the complete results, including r, raw *p*, FDR-adjusted *p*, and 95% confidence intervals, are provided in Table S2.

## 4. Discussion

This study examined inter-limb comparisons of isokinetic knee and hip variables and exploratory associations between these variables and video-derived sprint contact time. No statistically significant systematic differences were detected between the DL and NDL in PT, RPT, PP, RPP, or mean CT across sprint phases, and descriptive CT differences were small (approximately 0–3%). Two exploratory knee-extension comparisons at 240°/s yielded nominal raw *p* values below 0.05 for JAPT and TTPT, but neither remained statistically significant after correction across the 16 TTPT/JAPT comparisons. After correction for multiple testing, four sprint-time–CT correlations remained statistically significant, all involving NDL CT, whereas no isokinetic–CT correlation remained statistically significant. The nominal isokinetic findings are therefore interpreted as exploratory (Table 4).

The four sprint-time–CT associations that remained statistically significant after FDR correction involved NDL CT during the 5–20 m, 10–30 m, 30–60 m, and 45–60 m intervals. As expected, the highest interval-average velocity was observed over the 30–45 m interval, accompanied by decreased ground CT and increased FT (Figure 1). Although the corresponding DL coefficients did not remain statistically significant after correction, the DL and NDL coefficients were not formally compared. The apparent limb-classified differences should therefore be interpreted descriptively rather than as evidence that the associations genuinely differed between limbs [46,47,48].

For the torque and power variables, a greater number of raw *p*-values below 0.05 was observed at 240°/s than at 60°/s, and several nominal associations involved NDL CT. However, correlation coefficients were not formally compared across angular velocities or limb classifications; therefore, this distribution should be regarded as a descriptive numerical pattern rather than evidence that the correlations at 240°/s were statistically stronger than those at 60°/s. The interpretation is further limited by the substantial differences between open-chain isokinetic testing and sprint ground contact [49,50,51]. Moreover, none of the 832 torque–CT or 832 power–CT correlations remained statistically significant after FDR correction. Consequently, the present findings do not provide statistically robust evidence that high-velocity isokinetic torque or power is associated with ipsilateral or contralateral CT. The nominal patterns reported in Supplementary File require confirmation in larger, independently powered studies [28,52].

The TTPT and JAPT analyses were exploratory. Although 31 of the 832 TTPT/JAPT–CT correlations had raw *p*-values below 0.05, none remained statistically significant after FDR correction. The nominal findings included associations of knee JAPT and hip TTPT at 240°/s with NDL CT, but these results cannot be interpreted as statistically robust evidence of relationships between force-production timing and contralateral contact-time behavior. Without direct measurements of rate of torque development, muscle activation, joint kinetics, ground reaction forces, or intermuscular coordination, no underlying neuromuscular or mechanical mechanism can be inferred [17,53,54,55,56].

Some nominal hip TTPT correlations involved NDL CT during the early acceleration intervals, but these associations did not survive FDR correction. Habitual sprint-training practices, including block-start mechanics and hurdle clearance, may nevertheless warrant consideration in future confirmatory work [52,57,58,59]. Because block position was not included as a planned analytical factor and functional dominance during sprinting was not independently established, its potential influence on the nominal correlation patterns cannot be determined [59,60,61]. Future studies should record and model block position, starting preference, and task-specific limb dominance.

Several limitations should be considered when interpreting the present findings. The primary limitation is the absence of a formal reliability and measurement-error assessment for the video-derived CT and FT measurements. Although all frame-by-frame analyses were performed by one experienced evaluator and uncertain touchdown and toe-off events were reviewed before final frame selection, these quality-control procedures do not replace formal estimates of intra-rater reliability and measurement error [43,44,62]. At 240 frames/s, one frame corresponds to approximately 4.17 ms; uncertainty at either event boundary may therefore be of a similar magnitude to the small descriptive inter-limb CT differences observed in this study (approximately 0–3%) and could be greater if both boundaries were affected. Consequently, it cannot be determined whether the observed differences exceeded the measurement error of the video-analysis procedure or reflected genuine inter-limb differences; likewise, the absence of statistically significant differences cannot be interpreted as evidence of bilateral equivalence.

Measurement uncertainty also affects the interpretation of the correlation analyses. FDR-adjusted *p*-values and confidence intervals address multiplicity and sampling uncertainty, respectively, but neither account for measurement error in CT, which may attenuate, inflate, or otherwise destabilize the observed associations, particularly in a small sample. Accordingly, the nominal limb-specific and cross-limb isokinetic associations should be regarded as exploratory and hypothesis-generating until confirmed using a prespecified reliability protocol and independent or repeated ratings. Future studies should quantify intra-rater and, where relevant, inter-rater reliability through repeated frame identification and report ICCs alongside absolute reliability indices such as typical error, standard error of measurement, coefficient of variation, and Bland–Altman limits of agreement. Reliability should also be examined across acceleration phases; where feasible, higher-frame-rate cameras, synchronized force platforms, instrumented tracks, or validated contact-detection systems should be used as reference methods.

In addition, CT and FT were derived only from each athlete’s fastest 60 m trial rather than averaged across the three trials. Although the trial was selected a priori according to total sprint time and before CT and FT were extracted, this performance-based selection may have influenced the values included in the correlation analyses and does not characterize within-athlete variability across repeated trials. The reported associations should therefore be interpreted as pertaining specifically to best-trial performance. Future studies should analyze all repeated trials using trial-averaged estimates or multilevel statistical approaches.

A second limitation is that isokinetic testing was performed under non-weight-bearing, open-chain conditions, which differ from the closed-chain, weight-bearing, multi-joint demands of sprinting. Although 240°/s was selected as the higher angular-velocity condition, it does not reproduce the extremely brief ground-contact periods or multiplanar mechanics of maximal sprinting. In addition, all participants completed the isokinetic protocol using the same testing order (dominant limb before non-dominant limb, knee before hip, and 60°/s before 240°/s). Although this standardized sequence ensured procedural consistency across participants, the fixed dominant-before-non-dominant limb order meant that possible fatigue, familiarization, or carryover effects were systematically confounded with limb classification. Similarly, the fixed knee-before-hip order may have influenced subsequent hip performance. The absence of statistically significant inter-limb differences does not exclude these potential order effects, which should therefore be considered when interpreting both the inter-limb comparisons and limb-specific associations. Third, limb dominance was defined according to self-reported kicking preference. This classification may not correspond to sprint-specific functional dominance, front-block preference, or the limb used preferentially for propulsion and stabilization. Block position and habitual training asymmetries were not analyzed as independent factors. Fourth, the sample was small and included athletes specializing in different sprint and hurdle events. The sensitivity analysis indicated that, with 19 participants and an alpha level of 0.05, the study had 80% power to detect only correlations of |ρ| ≥ 0.59. Consequently, moderate associations may not have reached statistical significance, and the non-significant findings, particularly those involving variables assessed at 60°/s, should not be interpreted as evidence that no relationship exists. Differences in event-specific training backgrounds and competitive demands may therefore have influenced the observed phase-specific associations and should be considered when interpreting the findings. Because several sprint intervals contained some of the same ground contacts, associations across overlapping intervals should not be interpreted as independent replications. This temporal redundancy, together with the large number of tests relative to the sample size, further supports an exploratory interpretation despite the inclusion of all eligible correlations in the FDR procedure. No isokinetic–CT association remained statistically significant after correction. The nominal unadjusted findings should therefore be regarded as exploratory and require replication in larger, discipline-specific cohorts. Finally, ground reaction forces, joint moments, EMG, ankle-foot strength, and stiffness variables were not measured. Consequently, the study cannot identify the biomechanical or neuromuscular mechanisms underlying the observed statistical associations.

## 5. Conclusions

No statistically significant systematic inter-limb differences were identified in PT, RPT, PP, RPP, or mean video-derived CT across the examined sprint phases. Two exploratory knee-extension comparisons at 240°/s yielded nominal raw *p* values below 0.05 for JAPT and TTPT, but neither remained statistically significant after Benjamini–Hochberg correction across the 16 temporal/angular comparisons. Four sprint-time–CT correlations remained statistically significant after FDR correction, all involving NDL CT. In contrast, none of the torque–CT, power–CT, or TTPT/JAPT–CT correlations remained statistically significant after correction. The nominal isokinetic findings should therefore be regarded as hypothesis-generating and do not establish genuine inter-limb differences, bilateral equivalence, or contralateral biomechanical influence. Future studies should incorporate prespecified reliability testing, higher-precision or independently validated measurement methods, larger samples, and direct kinetic and neuromuscular assessments.

From a practical perspective, the observed 0–3% CT differences should not be used as individual asymmetry thresholds without knowledge of the typical error and smallest detectable change in the measurement procedure [63]. Previous research has also emphasized the importance of measurement reliability when interpreting inter-limb asymmetries in athletic performance assessments [64]. Isokinetic and sprint-specific assessments may be used together, but individual longitudinal interpretation should rely on methods with established absolute reliability.

## Figures and Tables

**Table 1:** Cohen’s d_z_ effect sizes and 95% confidence intervals for the mean differences in the primary paired comparisons.

Joint	Movement	Angular Velocity	Variable	95% CI for Mean Difference	*p* Value	Cohen’s dz
Knee	Extension	60°/s	PT	−13.345 to 5.872	0.425	−0.187
Knee	Extension	60°/s	RPT	−0.183 to 0.080	0.418	−0.190
Knee	Extension	60°/s	PP	−7.018 to 7.018	1.000	0.000
Knee	Extension	60°/s	RPP	−0.097 to 0.091	0.945	−0.016
Knee	Flexion	60°/s	PT	−5.636 to 9.005	0.635	0.111
Knee	Flexion	60°/s	RPT	−0.075 to 0.118	0.647	0.107
Knee	Flexion	60°/s	PP	−3.287 to 8.340	0.373	0.209
Knee	Flexion	60°/s	RPP	−0.044 to 0.109	0.387	0.204
Knee	Extension	240°/s	PT	−5.480 to 2.006	0.343	−0.224
Knee	Extension	240°/s	RPT	−0.076 to 0.026	0.311	−0.239
Knee	Extension	240°/s	PP	−6.756 to 12.019	0.563	0.135
Knee	Extension	240°/s	RPP	−0.094 to 0.159	0.592	0.125
Knee	Flexion	240°/s	PT	−4.482 to 4.166	0.940	−0.018
Knee	Flexion	240°/s	RPT	−0.060 to 0.051	0.856	−0.042
Knee	Flexion	240°/s	PP	−5.844 to 13.423	0.419	0.190
Knee	Flexion	240°/s	RPP	−0.079 to 0.169	0.459	0.174
Hip	Extension	60°/s	PT	−11.068 to 7.699	0.711	−0.087
Hip	Extension	60°/s	RPT	−0.150 to 0.116	0.787	−0.063
Hip	Extension	60°/s	PP	−8.439 to 5.281	0.635	−0.111
Hip	Extension	60°/s	RPP	−0.117 to 0.080	0.695	−0.092
Hip	Flexion	60°/s	PT	−2.644 to 9.276	0.258	0.268
Hip	Flexion	60°/s	RPT	−0.036 to 0.132	0.246	0.275
Hip	Flexion	60°/s	PP	−4.571 to 5.202	0.894	0.031
Hip	Flexion	60°/s	RPP	−0.063 to 0.074	0.873	0.037
Hip	Extension	240°/s	PT	−8.128 to 8.338	0.979	0.006
Hip	Extension	240°/s	RPT	−0.111 to 0.114	0.972	0.008
Hip	Extension	240°/s	PP	−18.647 to 16.857	0.917	−0.024
Hip	Extension	240°/s	RPP	−0.250 to 0.233	0.941	−0.017
Hip	Flexion	240°/s	PT	−1.923 to 8.975	0.191	0.312
Hip	Flexion	240°/s	RPT	−0.027 to 0.120	0.198	0.307
Hip	Flexion	240°/s	PP	−10.044 to 9.202	0.928	−0.021
Hip	Flexion	240°/s	RPP	−0.138 to 0.116	0.859	−0.041

PT, peak torque; RPT, relative peak torque; PP, peak power; RPP, relative peak power; CI, confidence interval. Cohen’s *d_z_* values represent effect sizes for paired comparisons between the dominant and non-dominant limbs.

**Table 2:** Sprint-time–contact-time correlations remaining statistically significant after Benjamini–Hochberg correction.

Sprint Phase	CT Limb	*r*	Raw *p*-Value	FDR-Adjusted *p*-Value	95% CI
5–20 m	NDL	0.823	<0.001	<0.001	0.588 to 0.929
10–30 m	NDL	0.650	0.003	0.017	0.278 to 0.853
30–60 m	NDL	0.720	<0.001	0.004	0.395 to 0.885
45–60 m	NDL	0.749	<0.001	0.003	0.447 to 0.898

CT, contact time; NDL, non-dominant limb; CI, confidence interval; FDR, false discovery rate. Raw *p*-values correspond to the Pearson correlation coefficients reported in the table. FDR-adjusted *p*-values were obtained by entering all 26 sprint-time–CT correlations into the Benjamini–Hochberg procedure. Confidence intervals were calculated using Fisher’s z transformation.

**Table 3:** Summary of the Benjamini–Hochberg false discovery rate correction across correlation families.

FDR Family	Total Correlations	Raw *p* < 0.05	FDR-Adjusted *p* < 0.05
Sprint time–CT	26	11	4
Torque–CT	832	42	0
Power–CT	832	52	0
TTPT/JAPT–CT	832	31	0

CT, contact time; PT, peak torque; RPT, relative peak torque; PP, peak power; RPP, relative peak power; TTPT, time to peak torque; JAPT, joint angle at peak torque; FDR, false discovery rate. All eligible correlations within each family were included in the Benjamini–Hochberg procedure irrespective of their raw *p*-values.

**Table 4:** Summary of the main inter-limb comparisons and exploratory correlation results.

Domain	Main Finding
Sprint performance	Mean 60 m sprint time: 7.36 ± 0.20 s; highest interval-average velocity: 9.36 ± 0.35 m/s
Contact time comparison	No statistically significant DL–NDL differences were observed; descriptive differences were approximately 0–3%, but their magnitude relative to measurement error is unknown
Isokinetic torque comparison	No significant DL–NDL differences in PT or RPT at 60°/s or 240°/s
Isokinetic power comparison	No significant DL–NDL differences in PP or RPP
TTPT and JAPT comparison	Two nominal knee-extension differences at 240°/s (JAPT and TTPT) did not remain statistically significant after Benjamini–Hochberg correction across the 16 exploratory temporal/angular comparisons
Associations at 60°/s	Nominal unadjusted associations were observed, but no isokinetic–CT correlation remained statistically significant after FDR correction
Associations at 240°/s	A greater number of nominal associations was observed at 240°/s than at 60°/s; correlation coefficients were not formally compared between velocities, and none remained statistically significant after FDR correction.
Limb-classified correlations	Four sprint-time–CT correlations remained statistically significant after FDR correction, all involving NDL CT; DL and NDL coefficients were not formally compared
Cross-limb correlations	Nominal cross-limb isokinetic–CT associations did not remain statistically significant after FDR correction and do not establish contralateral influence

## Data Availability

Data available upon request.

## Supplementary Materials

The following supporting information can be downloaded at: https://www.mdpi.com/article/10.3390/life16101762/s1. Table S1: Complete Pearson correlation matrix with Benjamini–Hochberg FDR adjustment, Table S2. Exploratory paired inter-limb comparisons of time to peak torque and joint angle at peak torque, Table S3: Small-sample sensitivity analyses for the four FDR-significant Pearson sprint-time–CT associations.

## Abbreviations

The following abbreviations are used in this manuscript:

AI	Asymmetry Index
BW	Body Weight
CT	Contact Time
CV	Coefficient of Variation
DL	Dominant Limb
EMG	Electromyography
FT	Flight Time
ICC	Intraclass Correlation Coefficient
JAPT	Joint Angle at Peak Torque
NDL	Non-Dominant Limb
PP	Peak Power
PT	Peak Torque
ROM	Range of Motion
RPP	Relative Peak Power
RPT	Relative Peak Torque
SD	Standard Deviation
SPSS	Statistical Package for the Social Sciences
TE	Typical Error
TTPT	Time to Peak Torque
